# Dietary Nitrate Supplementation Improves Exercise Tolerance by Reducing Muscle Fatigue and Perceptual Responses

**DOI:** 10.3389/fphys.2019.00404

**Published:** 2019-04-24

**Authors:** Florian Husmann, Sven Bruhn, Thomas Mittlmeier, Volker Zschorlich, Martin Behrens

**Affiliations:** ^1^Institute of Sport Science, University of Rostock, Rostock, Germany; ^2^Department of Traumatology, Hand and Reconstructive Surgery, University Medicine Rostock, Rostock, Germany

**Keywords:** beetroot juice, central fatigue, contractile function, muscle pain, performance fatigability, peripheral fatigue

## Abstract

The present study was designed to provide further insight into the mechanistic basis for the improved exercise tolerance following dietary nitrate supplementation. In a randomized, double-blind, crossover design, twelve recreationally active males completed a dynamic time-to-exhaustion test of the knee extensors after 5 days of consuming both nitrate-rich (NITRATE) and nitrate-depleted beetroot juice (PLACEBO). Participants who improved their time-to-exhaustion following NITRATE performed a time-matched trial corresponding to the PLACEBO exercise duration with another 5 days of dietary nitrate supplementation. This procedure was performed to obtain time-matched exercise trials with (NITRATE_tm_) and without dietary nitrate supplementation (PLACEBO). Neuromuscular tests were performed before and after each time-matched condition. Muscle fatigue was quantified as percentage change in maximal voluntary torque from pre- to post-exercise (ΔMVT). Changes in voluntary activation (ΔVA) and quadriceps twitch torque (ΔPS100) were used to quantify central and peripheral factors of muscle fatigue, respectively. Muscle oxygen saturation, quadriceps muscle activity as well as perceptual data (i.e., perception of effort and leg muscle pain) were recorded during exercise. Time-to-exhaustion was improved with NITRATE (12:41 ± 07:18 min) compared to PLACEBO (09:03 ± 04:18 min; *P* = 0.010). NITRATE_tm_ resulted in both lower ΔMVT and ΔPS100 compared to PLACEBO (*P* = 0.002; *P* = 0.001, respectively). ΔVA was not different between conditions (*P* = 0.308). NITRATE_tm_ resulted in reduced perception of effort and leg muscle pain. Our findings extend the mechanistic basis for the improved exercise tolerance by showing that dietary nitrate supplementation (i) attenuated the development of muscle fatigue by reducing the exercise-induced impairments in contractile muscle function; and (ii) lowered the perception of both effort and leg muscle pain during exercise.

## Introduction

The capacity to maintain physical activity (i.e., exercise tolerance) is crucial for endurance athletes, but is at least as important for the general population since it has been shown that poor aerobic fitness is linked to cardiovascular disease and overall mortality (Myers et al., 2002; Kodama et al., 2009). Exercise intolerance is known as a major symptom of various diseases [e.g., peripheral arterial disease (Leeper et al., 2013), chronic obstructive pulmonary disease (Pepin et al., 2007), or chronic heart failure (Mentzer and Auseon, 2012) with detrimental consequences for quality of life (Belfer and Reardon, 2009)]. It is therefore not surprising that its underpinning mechanisms have been extensively investigated for more than a century (McKenna and Hargreaves, 2007), but are still highly debated (Marcora and Staiano, 2010). As a multifactorial phenomenon, exercise tolerance is determined by various physiological [e.g., cardiovascular, respiratory, metabolic, and neuromuscular mechanisms (McKenna and Hargreaves, 2007)] and psychological factors [e.g., external motivation, mental fatigue (McCormick et al., 2015)]. Given its broad significance, many efforts have been made to identify possible interventions to improve exercise tolerance by utilizing, e.g., nutritional (Matson and Tran, 1993), pharmacological (Mauger et al., 2014), or psychological strategies (McCormick et al., 2015).

Dietary nitrate supplementation, commonly administered in the form of beetroot juice, has been demonstrated as a promising approach to improve exercise tolerance at low and high intensities (Bailey et al., 2009, 2010; Lansley et al., 2011; Thompson et al., 2014). The ergogenic effect of dietary nitrate on exercise tolerance is attributed to the actions of the biological messenger nitric oxide (NO), since dietary nitrate supplementation is thought to be an effective method to elevate its bioavailability (Lundberg and Govoni, 2004). NO is known for its regulatory function in various physiological processes including vasodilation (Ignarro, 1989), angiogenesis (Papapetropoulos et al., 1997), mitochondrial respiration (Brown, 1995), and contractile function (Kobzik et al., 1994). Several studies have shown that the ergogenic effect of dietary nitrate on exercise tolerance is associated with lower oxygen (O_2_) cost of submaximal exercise (Bailey et al., 2009; Larsen et al., 2010, 2011), which might be related to a more efficient mitochondrial adenosine triphosphate (ATP) synthesis (Larsen et al., 2011) and/or a more efficient ATP utilization during skeletal muscle work (Bailey et al., 2010). Moreover, increased dietary nitrate intake has been shown to improve vascular (Ferguson et al., 2013), metabolic (Bailey et al., 2010), and skeletal muscle function in response to exercise (Hernández et al., 2012). Any of the physiological alterations associated with dietary nitrate are capable of modulating skeletal muscle function (Affourtit et al., 2015) and thus the development of muscle fatigue. Muscle fatigue [also referred to as ‘performance fatigability’ (Enoka and Duchateau, 2016)] is characterized by impairments in motor performance resulting from an exercise-induced decline in the force-generating capacity of the involved muscles and stems from a decrease in neural activation of muscles (traditionally termed ‘central fatigue’) and/or alterations at or distal to the neuromuscular junction that result in contractile dysfunction (traditionally termed ‘peripheral fatigue’) (Gandevia, 2001). The capacity of the neuromuscular system to generate the required power for the task is considered as critical factor of endurance performance (Burnley and Jones, 2018). However, the traditional assumption that exercise tolerance is exclusively limited by the inability to generate the power output required for the task despite maximal voluntary effort (also referred to as ‘task failure’) is highly debated (Enoka and Duchateau, 2016). Several authors suggest that endurance performance is rather regulated by a complex interplay of physiological and psychological factors (Marcora, 2008; Noakes, 2008; Venhorst et al., 2018). Particularly effort perception and muscle pain are considered as important factors that determine exercise tolerance (Noakes, 2008; Marcora and Staiano, 2010; Mauger, 2014).

To the authors’ knowledge, no study to date has investigated the impact of dietary nitrate supplementation on central and peripheral mechanisms of muscle fatigue or its impact on effort and muscle pain perception during submaximal endurance exercise. The present study was designed to provide further insight into the mechanistic basis for the improved exercise tolerance after dietary nitrate supplementation by investigating key-determinants of endurance performance. Therefore, we quantified exercise tolerance via the use of single-joint endurance exercise, which provides a suitable model to investigate the underlying mechanisms of endurance performance without cardiorespiratory limitations typically associated with whole-body endurance exercise (Andersen et al., 1985). First, by using a randomized, counterbalanced, double-blind, crossover design, participants completed a high-intensity time-to-exhaustion test of the knee extensors after 5 days of dietary nitrate and placebo supplementation. Second, those who improved their time-to-exhaustion with dietary nitrate, performed a time-matched trial corresponding to the exercise duration of the placebo condition. The time-matched conditions were further examined to analyze the impact of dietary nitrate on (i) central and peripheral aspects of muscle fatigue; (ii) muscle O_2_ saturation (SmO_2_), (iii) electromyographic (EMG) activity, and (iv) perception of effort and leg muscle pain. To improve the validity of the present data, we also aimed to control for distinct factors (e.g., task motivation, trait and state fatigue), which are thought to affect both performance and perceptual measures during fatiguing exercise (Pageaux, 2014; Enoka and Duchateau, 2016).

We hypothesized that muscle fatigue development is attenuated with dietary nitrate supplementation.

## Materials and Methods

### Subjects

An *a priori* sample size calculation was conducted based on the effect size of a previously published study investigating, amongst others, the impact of dietary nitrate on time-to-exhaustion during high-intensity knee extension exercise (Bailey et al., 2010). A two-sided significance level of 0.05 and a power of 0.95 indicated that 8 participants would be required. Based on the observation that not all participants improved their exercise tolerance following dietary nitrate supplementation (Wilkerson et al., 2012; Coggan et al., 2018), 14 recreationally active males were initially recruited to participate in the present study. Given the fact that two participants did not reach exhaustion within the predefined time window (25 min) of the fatiguing task, a total of 12 subjects (age: 27 ± 5 years; height: 183 ± 7 cm; body mass: 85 ± 9 kg; physical activity: 6 ± 3 h ⋅ wk^-1^) was considered for analysis. Taking into account that muscle fatigue is thought to depend on sex [for a review see (Hunter, 2014)], we chose a sample comprising exclusively male subjects. All participants were familiar with high-intensity exercise. Subjects were asked to abstain from (i) vigorous exercise, analgesics, caffeine, and alcohol consumption for 24 h prior to the laboratory visits as well as (ii) nitrate-rich foods [i.e., leafy green vegetables, beetroot, and processed meats (Hord et al., 2009)] and (iii) antibacterial mouthwash during the entire study period (Bondonno et al., 2015). Furthermore, participants were instructed to record their diet 24 h prior to the first laboratory visit and to repeat this for all subsequent visits. The study was approved by the university ethics committee and was conducted according to the Declaration of Helsinki. All participants gave written informed consent in accordance with the Declaration of Helsinki.

### Experimental Procedure

Subjects visited the laboratory on at least three different occasions. During the first visit, participants were thoroughly familiarized with the following procedures: (i) one-leg dynamic exercise (OLDE) of the knee extensors (for more details, see *One-leg dynamic exercise*); (ii) neuromuscular tests comprising maximal voluntary contractions (MVC), and peripheral nerve stimulation as well as (iii) ratings of perceived effort and leg muscle pain. Furthermore, an OLDE incremental test was performed to determine peak power output.

**FIGURE 1:**
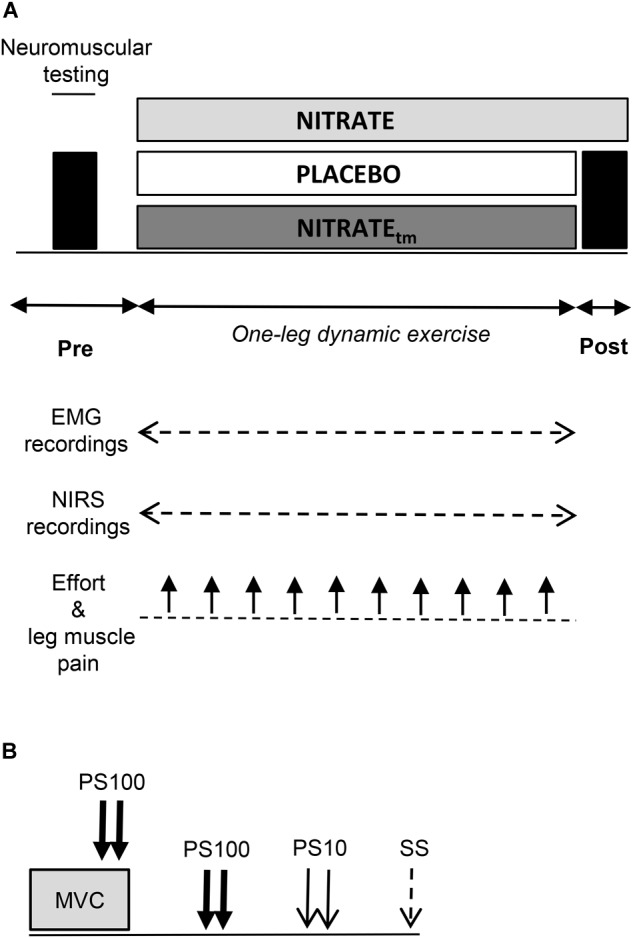
**(A)** Illustration of the experimental design. A time-to-exhaustion test of the knee extensors was performed after 5 days of dietary nitrate (NITRATE) and PLACEBO supplementation. Neuromuscular function of the quadriceps muscle was assessed before and immediately after both PLACEBO and time-matched dietary nitrate condition (NITRATE_tm_). Effort perception and leg muscle pain were recorded every min during exercise. Electromyography (EMG) and near-infrared spectroscopy (NIRS) data were continuously recorded during exercise. **(B)** The neuromuscular testing procedure comprised isometric MVC of the knee extensors combined with electrical stimulation to assess maximal voluntary torque (MVT), voluntary activation (via the interpolated twitch technique), and quadriceps twitch torques in response to paired electrical stimuli at 100 Hz (PS100) and at 10 Hz (PS10) as well as single stimuli (SS). A representative torque-time curve of the neuromuscular assessment procedure can be found in a previous publication from our group (Husmann et al., 2018).

Using a randomized, counterbalanced, double-blind, crossover design, participants performed an OLDE time-to-exhaustion test of the knee extensors at 85% peak power output after 5 days of supplementation with dietary nitrate via beetroot juice (NITRATE; ∼6.5 mmol nitrate per 70 mL; Beet it, Heartbeet Ltd., Ipswich, United Kingdom) and nitrate-depleted beetroot juice (PLACEBO; ∼0.04 mmol nitrate per 70 mL; Beet it, Heartbeet Ltd., Ipswich, United Kingdom), respectively. The duration of the supplementation period was chosen based on data from Bailey et al. (2010), who have shown that time-to-exhaustion during high-intensity knee extension exercise was significantly improved following 4–6 days of dietary nitrate supplementation. For each experimental condition, subjects were instructed to consume 70 mL beetroot juice every morning and 2 h prior to the laboratory visits. The second and third occasion was separated by 7 ± 1 days and took place at the same time of the day (±2 h). Participants who improved their time-to-exhaustion following NITRATE by at least 8% performed a time-matched trial corresponding to the PLACEBO exercise duration after a 10 days wash-out period (Larsen et al., 2007) and another 5 days of dietary nitrate supplementation. This procedure was performed in order to allow comparison of neuromuscular, oxygenation, and perceptual data between time-matched exercise trials with (NITRATE_tm_) and without dietary nitrate supplementation (PLACEBO). Time-to-exhaustion tests that do not differ by more than 8% between conditions were considered as time-matched. The worthwhile change for the time-to-exhaustion test was defined according to Pageaux et al. (2015) who reported a coefficient of variation of ∼8% for the intersession reliability of the OLDE protocol. Neuromuscular tests were performed before and immediately after exercise termination (<10 s) (Figure 1A). SmO_2_ and EMG data as well as ratings of perceived effort and leg muscle pain were continuously recorded during each experimental condition.

Prior to pre-exercise measurements, participants performed an initial warm-up on a stationary bicycle (5 min; 100 W; 90 rpm) followed by a specific warm-up on a dynamometer comprising two isometric contractions for 5 s at 50, 70, and 90% of maximal voluntary torque interspaced by 60 s of rest (MVT; determined during the familiarization session), respectively. Neuromuscular tests comprised supramaximal electrical stimulations of the femoral nerve during and after an isometric MVC (Figure 1B). All measurements were conducted on the quadriceps muscle of the dominant leg (i.e., kicking preference). During OLDE and neuromuscular testing, subjects were comfortably seated and secured on a CYBEX NORM dynamometer (Computer Sports Medicine^®^, Inc., Stoughton, MA, United States). The seating position was adjusted for each participant and settings were documented for the subsequent sessions.

### One-Leg Dynamic Exercise

One-leg dynamic exercise is an exercise protocol characterized by rhythmic isotonic contractions of the knee extensor muscles alternated with passive knee flexions. In contrast to whole body exercise, OLDE is not limited by cardiorespiratory function (Rossman et al., 2014). The OLDE protocol used in the present study was recently developed and proved as reliable to measure muscle endurance and to investigate central and peripheral mechanisms of muscle fatigue (Pageaux et al., 2015). It has been shown that OLDE induces severe levels of muscle fatigue (-40% MVT) with significant impairments in both peripheral (-40% contractile twitch torque) and central factors (-13% voluntary activation) (Pageaux et al., 2015). Furthermore, it allows bypassing the time lag between exercise termination and neuromuscular testing, which is typically associated with whole body endurance exercise. A more detailed description of the OLDE protocol used in the present study can be found elsewhere (Pageaux et al., 2015). Briefly, OLDE was performed on a CYBEX NORM dynamometer (Computer Sports Medicine^®^, Inc., Stoughton, MA, United States) with the range of motion set from 10 to 90° (0° = full knee extension). A metronome was used to ensure a cadence of 50 contractions per min (cpm), enabling an active knee extension with ∼106°⋅ s^-1^ and a passive knee flexion velocity of ∼180°⋅ s^-1^.

During the first visit, subjects were thoroughly familiarized with the OLDE protocol using torque and EMG feedback. An OLDE incremental test was performed afterwards to determine peak power output (89.5 ± 13.1 W). Testing started at an isotonic load of 4 N ⋅ m (∼7.4 W) for 1 min and was increased by 3 N ⋅ m every min (∼4.5 W) until exhaustion. Exhaustion was defined as a decline in cadence below 40 cpm for a period of ≥10 s despite strong verbal encouragement. On the second and third visit, subjects performed a 2 min warm-up at 10% peak power output followed by a high-intensity OLDE time-to-exhaustion test at 85% of peak power output (76.0 ± 11.1 W). Monetary rewards were announced for the three best performances (50 €, 30 €, and 20 €) in order to motivate the participants to exercise for as long as possible during the time-to-exhaustion test. Exhaustion was again defined as a drop in cadence below 40 cpm for a period of ≥10 s despite strong verbal encouragement. An upper time limit for the time-to-exhaustion test was set at 25 min.

### Torque Recordings

A CYBEX NORM dynamometer (Computer Sports Medicine^®^, Inc., Stoughton, MA, United States) was used to capture instantaneous torques. Participants were seated on an adjustable chair with the hip fixed at 80° (0° = full extension). Straps were fixed tightly across the subjects’ waist and chest to avoid excessive movements during data recording. The dynamometer rotation axis was aligned with the knee joint rotation axis and the lever arm was attached to the lower leg just above the lateral malleolus. Isometric MVC were performed at 90° knee flexion (0° = full extension). Isometric MVT was defined as the highest torque value prior to the superimposed twitch evoked by electrical stimulation. For each trial, subjects were instructed to cross their arms in front of their chest and to push as hard and as fast as possible against the lever arm of the dynamometer. Strong verbal encouragement was given by the investigator during MVC testing. Visual feedback of the torque-time curve was provided on a digital oscilloscope (HM1508, HAMEG Instruments, Mainhausen, Germany).

### EMG Recordings

A detailed description of the EMG recordings can be found in a previously published study from our laboratory (Behrens et al., 2015). Briefly, myoelectrical signals of the vastus medialis (VM), rectus femoris (RF), and vastus lateralis (VL) muscles were recorded using surface electrodes in a bipolar configuration (EMG Ambu Blue Sensor N). EMG signals were amplified (2500×), band-pass filtered (10–450 Hz), and digitized with a sampling frequency of 3 kHz using an analog-to-digital converter (NI PCI-6229, National Instruments, Austin, TX, United States). Maximum compound muscle action potential amplitudes (*M*_max_) elicited by electrical stimulation were measured peak-to-peak. Muscle activity during exercise was assessed by calculating the root mean square of the EMG signal (RMS-EMG) averaged for five contractions at the beginning, as well as at 25, 50, 75, and 100% of each trial, respectively. Only EMG data during the concentric phase of each repetition were considered for analysis. RMS-EMG of VM, RF, and VL was normalized to the corresponding *M*_max_ value (RMS ⋅ M^-1^). To estimate the total muscle activity of the quadriceps during knee-extension exercise, RMS ⋅ M^-1^ was averaged across VM, RF, and VL (Husmann et al., 2017).

### Electrical Nerve Stimulation

Neuromuscular function of the quadriceps muscle was assessed by using electrical stimulation of the femoral nerve. A constant-current stimulator (Digitimer DS7A, Hertfordshire, United Kingdom) was used to deliver square-wave pulses of 1 ms duration with maximal voltage of 400 V. A ball probe cathode (10 mm diameter) was pressed in the femoral triangle always by the same experienced investigator. The anode, a self-adhesive electrode (35 mm × 45 mm, Spes Medica, Genova, Italy), was affixed over the greater trochanter. After determining the optimal site for stimulation, the position was marked onto the subjects’ skin to ensure repeatable measurements within each session. Individual stimulation intensity was progressively increased until *M*_max_ of VM, RF, and VL as well as a plateau in knee extensor twitch torque was achieved. During the subsequent testing procedures, the stimulation intensity was increased by additional 40% to guarantee supramaximal stimulation. Potentiated quadriceps twitch torques evoked by paired electrical stimuli at 100 Hz (PS100), 10 Hz (PS10), and single stimuli (SS) were elicited 2, 4, and 6 s after isometric MVC, respectively. Peak twitch torques (i.e., highest values of the torque-time curve) were determined for PS100, PS10, and SS, respectively. The PS10 ⋅ PS100^-1^ torque ratio was calculated as an index of low-frequency fatigue. A reduction of this ratio is thought to indicate impairments in excitation-contraction coupling (Verges et al., 2009). To determine the level of voluntary activation during isometric MVC, the interpolated twitch technique was applied. Electrical paired stimuli were delivered to the femoral nerve at 90° knee flexion 2 s after torque onset (during the plateau phase) and 2 s after MVC. The level of voluntary activation was calculated using a corrected formula: [1 - superimposed twitch (*T*_b_ × MVT^-1^) × control twitch^-1^] × 100 (Strojnik and Komi, 1998). MVT is the maximal torque level and *T*_b_ the torque value immediately before the superimposed twitch. The corrected formula is used to avoid the potential problem that the superimposed stimuli are not always applied during the maximum torque level. As shown recently by our group, voluntary activation of the knee extensors can be reliably assessed during isometric contractions using the corrected formula (Behrens et al., 2017).

### Muscle Oxygenation

Muscle oxygenation of VL was continuously monitored using a portable near-infrared spectroscopy (NIRS) device (Moxy, Fortiori Design LLC, Hutchinson, MN, United States). The Moxy monitor has been recently shown to allow reliable measurements of SmO_2_ (Crum et al., 2017). SmO_2_ reflects the balance between O_2_ delivery and O_2_ demand in the analyzed muscle (Ferrari et al., 2011). Prior to optode placement on the VL, subjects’ skin was shaved and cleaned. The NIRS probe was attached at mid-thigh level, closely to the VL EMG electrodes. The optode was secured with tape and covered with a protective shell to avoid artifacts caused by motion and light. Reliable optode placement between sessions was assured by recording the distance to the patella, measured from the subject’s patella to the greater trochanter. Furthermore, skinfold thickness above the VL was measured using a skinfold caliper (5 ± 1 mm). All signals were recorded with a sampling frequency of 2 Hz. A 4th order low-pass zero-phase Butterworth filter (cutoff frequency 0.2 Hz) was applied. NIRS-derived indices of muscle oxygenation were averaged across 5 s at 25, 50, 75, and 100% of each trial, respectively. Shortly after the OLDE warm-up, resting baseline values were averaged for 30 s prior to the start of the OLDE protocol. Baseline values were captured at rest in a seated position. SmO_2_ and total hemoglobin (tHb) were reported as percentage changes from baseline (ΔSmO_2_ and ΔtHb).

### Ratings of Perceived Effort and Leg Muscle Pain

During the first visit at the laboratory, subjects were familiarized with ratings of perceived effort and ratings of leg muscle pain. Subjects’ perception of effort was recorded by using the 15-point Borg scale (Borg, 1982). Prior to each testing session, participants received written instructions based on guidelines recently proposed by Pageaux (2016). Briefly, instructions comprised the definition of effort (“the conscious sensation of how hard, heavy, and strenuous a physical task is”), exercise-specific descriptions (“How hard is it for you to drive your leg?”), exercise-anchoring (e.g., “maximal exertion corresponds to the effort you experienced while you were performing a MVC”), and the distinction of effort, pain, and other exercise-related sensations (Pageaux, 2016). Leg muscle pain was defined as the intensity of pain perceived by the subject exclusively in the exercising quadriceps. A modified category-ratio 10 (CR-10) scale was used to quantify leg muscle pain during exercise (Cook et al., 1997). At the beginning of each minute, the participants were asked to rate their perceived effort and leg muscle pain. The average of all ratings across the entire exercise duration is reported as mean levels of effort and leg muscle pain. End-exercise levels of effort and leg muscle pain refer to the last rating of effort and leg muscle pain before exercise termination.

### Task Motivation

Participants’ motivation to successfully complete the time-to-exhaustion test was assessed by using the success motivation and task interest motivation subscales designed and validated by Matthews et al. (2001). On a 5-point Likert scale (0 = not at all, 1 = a little bit, 2 = somewhat, 3 = very much, 4 = extremely) subjects rated 8 items (e.g., “I wanted to succeed on the task” and “I was eager to do well”). Therefore, total scores range between 0 and 32. The questionnaire was presented to the participant prior to the start of each task. In order to control for potential differences in task motivation, total scores were compared across all experimental conditions. If there is a significant difference in task motivation between conditions, it is considered as a covariate in the statistical analysis.

### Trait and State Properties of Fatigue

According to Enoka and Duchateau (2016), fatigue is defined as a disabling symptom which can be assessed by self-report and quantified as a state variable or as a trait characteristic. Both properties of fatigue are considered as modulating factors of human performance. The Modified Fatigue Impact Scale (MFIS), a self-reported measure of the impact of fatigue on cognitive, physical, and psychosocial aspects of daily activity, was utilized to assess the level of trait fatigue over the course of the last 7 days before each laboratory visit. State fatigue was examined by using the fatigue scale of the Profile of Mood States (POMS-F), which has been shown to provide a reliable and valid instrument to assess the level of state fatigue across a wide range of cohorts (O’Connor, 2004). Before each exercise trial, subjects were asked to complete both questionnaires. If there are significant differences in self-reported measures of fatigue between conditions, they are considered as covariates in the statistical analysis.

### Quantification of Muscle Fatigue

In the present study, muscle fatigue was quantified via the percentage change in MVT values from pre- to post-exercise (ΔMVT). Percentage changes in voluntary activation (ΔVA) and PS100 (ΔPS100) from pre- to post-exercise were used to quantify central and peripheral factors of muscle fatigue, respectively.

### Statistical Analysis

All data were screened for normal distribution using the Shapiro–Wilk test. Differences in percentage changes from pre- to post-exercise of all neuromuscular parameters were tested using Student’s paired *t*-tests. Cohen’s *d* effect size was calculated for each paired comparison. Effect sizes of 0.20, 0.50, and 0.80 were considered small, medium, and large, respectively (Cohen, 1988). Two-way ANOVAs with repeated measures on time and condition were conducted for all parameters derived from EMG and NIRS recordings during exercise. *Post hoc* tests were performed with Bonferroni adjustments. The effect size was determined by calculating partial eta squared ($\eta_{p}^{2}$). Data were analyzed using the SPSS statistical package 22.0 (SPSS Inc., Chicago, IL, United States) and statistical significance was accepted at *P* ≤ 0.05. Sample size was calculated with the statistical software package G^∗^Power (version 3.1.4.).

## Results

### Task Motivation

No differences in task motivation were found between PLACEBO (28 ± 3) and NITRATE (27 ± 3, *P* = 0.156). Furthermore, task motivation was not significantly different between time-matched conditions (PLACEBO: 26 ± 3; NITRATE_tm_: 27 ± 5; *P* = 0.282).

### Trait and State Properties of Fatigue

No differences in trait fatigue were observed between PLACEBO (12 ± 7) and NITRATE (11 ± 9, *P* = 0.324). Furthermore, trait fatigue was not significantly different between time-matched conditions (PLACEBO: 10 ± 6; NITRATE_tm_: 9 ± 7, *P* = 0.282). No differences in state fatigue were found between PLACEBO (8 ± 7) and NITRATE (7 ± 6, *P* = 0.386). Furthermore, state fatigue was not significantly different between time-matched conditions (PLACEBO: 8 ± 6; NITRATE_tm_: 7 ± 8, *P* = 0.346).

### Exercise Tolerance

**FIGURE 2:**
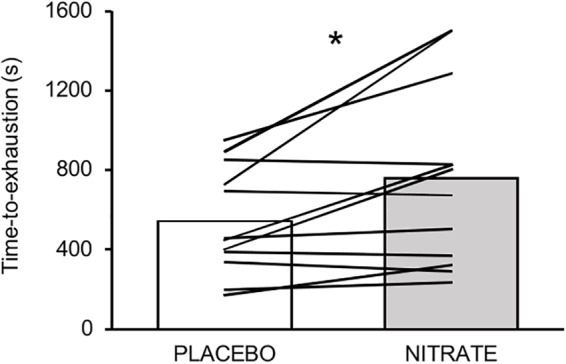
Mean values and individual data for time-to-exhaustion (s) between experimental conditions. Please note that eight out of twelve participants improved their performance over a range from ∼9 to ∼51%. Significantly different between conditions: ^∗^*P* ≤ 0.05.

Time-to-exhaustion was significantly improved with NITRATE (12:41 ± 07:18 min) compared to PLACEBO (09:03 ± 04:18 min, *P* = 0.010, *d* = 0.61). Individual data are presented in Figure 2. Eight participants improved their exercise performance following dietary nitrate supplementation by at least 8% and completed another trial corresponding to the PLACEBO exercise duration. Exercise trials that do not differ by more than 8% from each other were considered as time-matched. Together, time-matched conditions of 11 subjects were taken into account for further analysis and are referred to as NITRATE_tm_ and PLACEBO. Please note that two participants reached the upper time limit of 25 min during the NITRATE condition.

### Maximal Voluntary Torque

**FIGURE 3:**
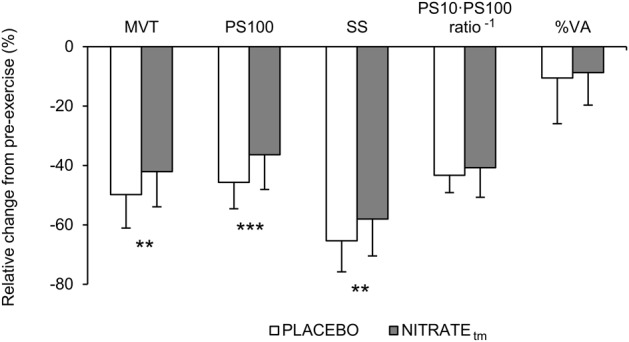
Percentage change from pre-exercise values for maximal voluntary torque (MVT), twitch torque in response to paired (PS100) and single (SS) electrical stimuli, PS10 ⋅ PS100^-1^ ratio, and voluntary activation (%VA) across both PLACEBO and time-matched dietary nitrate condition (NITRATE_tm_). Values are presented as mean ± SD. Significantly different between conditions: ^∗∗^*P* ≤ 0.01, ^∗∗∗^*P* ≤ 0.001.

A significantly lower ΔMVT was found for NITRATE_tm_ compared to PLACEBO (*P* = 0.002, *d* = 0.66; Figure 3). Absolute values for MVT are presented in Table 1.

**Table 1:** Neuromuscular function of the quadriceps muscle before and after each time-matched condition (*n* = 11).

		Pre	Post
MVT (N ⋅ m)			
	*PLACEBO*	304.7 ± 68.8	152.3 ± 47.4
	*NITRATE_tm_*	300.6 ± 61.0	175.0 ± 55.3
PS100 (N ⋅ m)			
	*PLACEBO*	107.2 ± 24.1	57.6 ± 11.6
	*NITRATE_tm_*	103.0 ± 21.4	65.0 ± 15.3
PS10 (N ⋅ m)			
	*PLACEBO*	103.9 ± 20.5	31.6 ± 6.8
	*NITRATE_tm_*	103.6 ± 18.4	39.3 ± 13.2
SS (N ⋅ m)			
	*PLACEBO*	69.1 ± 16.6	23.3 ± 5.0
	*NITRATE_tm_*	68.8 ± 16.4	28.3 ± 8.8
PS10 ⋅ PS100^-1^ ratio			
	*PLACEBO*	0.97 ± 0.05	0.55 ± 0.05
	NITRATE*_tm_*	1.01 ± 0.06	0.60 ± 0.10
VM *M*_max_ (mV)			
	*PLACEBO*	12.5 ± 3.3	12.5 ± 3.3
	*NITRATE_tm_*	13.2 ± 2.1	13.6 ± 2.2
RF *M*_max_ (mV)			
	*PLACEBO*	4.5 ± 2.1	4.0 ± 1.7
	*NITRATE_tm_*	4.2 ± 1.7	3.9 ± 1.7
VL *M*_max_ (mV)			
	*PLACEBO*	9.9 ± 3.6	10.0 ± 3.7
	*NITRATE_tm_*	9.3 ± 3.0	10.0 ± 3.4
VA (%)			
	*PLACEBO*	95.8 ± 2.8	85.9 ± 15.2
	*NITRATE_tm_*	95.6 ± 3.6	87.3 ± 10.7

*Values are expressed as mean ± SD. MVT, maximum voluntary torque; PS100, paired stimuli twitch torque at 100 Hz; PS10, paired stimuli twitch torque at 10 Hz; SS, single stimulus twitch torque; PS10 ⋅ PS100^-*1*^ ratio, index of low-frequency fatigue; M_*max*_, maximum M-wave; VM, vastus medialis; RF, rectus femoris; VL, vastus lateralis; VA, voluntary activation; NITRATE_*tm*_, time-matched dietary nitrate condition*.

### Electrically Evoked Twitch Torque

A significantly lower ΔPS100 was found for NITRATE_tm_ compared to PLACEBO (*P* = 0.001, *d* = 0.91; Figure 3). Furthermore, ΔSS was shown to be lower for NITRATE_tm_ compared to PLACEBO (*P* = 0.007, *d* = 0.64). No significant differences for ΔPS10 ⋅ PS100^-1^ ratio were found between NITRATE_tm_ and PLACEBO (*P* = 0.183, *d* = 0.31; Figure 3). Absolute values for PS100, SS, and PS10 ⋅ PS100^-1^ ratio are presented in Table 1.

### Electrically Evoked Potentials

No significant differences in Δ*M*_max_ between NITRATE_tm_ and PLACEBO were observed for VM, RF, and VL (*P* = 0.215, *d* = 0.37; *P* = 0.297, *d* = 0.16; *P* = 0.448, *d* = 0.03, respectively). Absolute values for *M*_max_ are presented in Table 1.

### Voluntary Activation

No significant differences between NITRATE_tm_ and PLACEBO were found for ΔVA (*P* = 0.308, *d* = 0.14; Figure 3). Absolute values for voluntary activation are presented in Table 1.

### EMG Recordings During Exercise

**FIGURE 4:**
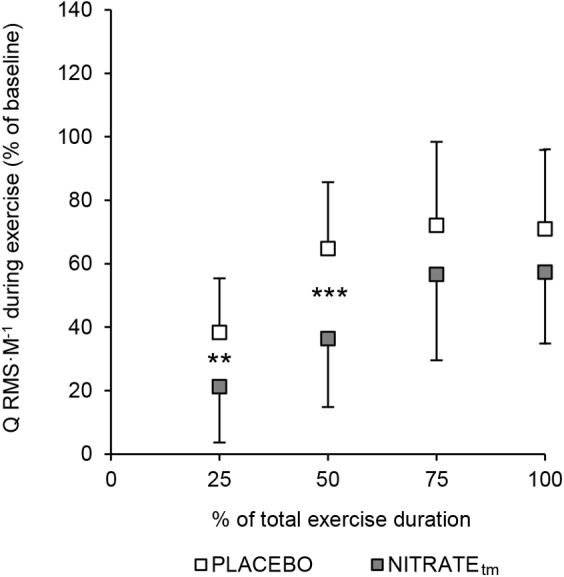
Percentage increase from baseline for the normalized muscle activity of the quadriceps muscle (Q RMS ⋅ M^-1^). To estimate the total muscle activity of the quadriceps muscle, RMS ⋅ M^-1^ was averaged across vastus medialis, rectus femoris, and vastus lateralis for five contractions at 25, 50, 75, and 100% of each trial, respectively. Baseline was defined as the average of the first five contractions. Values are presented as mean ± SD. NITRATE_tm_, time-matched dietary nitrate condition; RMS ⋅ M^-1^, the root mean square of the EMG signal normalized to *M*_max_. Significantly different between conditions: ^∗∗^*P* ≤ 0.01, ^∗∗∗^*P* ≤ 0.001.

A significant condition effect was shown for RF ΔRMS ⋅ M^-1^ (*F*_1,10_ = 6.85, *P* = 0.026, $\eta_{p}^{2}$ = 0.41) and VL ΔRMS ⋅ M^-1^ (*F*_1,10_ = 5.56, *P* = 0.040, $\eta_{p}^{2}$ = 0.36), but not for VM ΔRMS ⋅ M^-1^ (*F*_1,10_ = 2.34, *P* = 0.157, $\eta_{p}^{2}$ = 0.20). For RF ΔRMS ⋅ M^-1^, significant differences between PLACEBO and NITRATE_tm_ were found at 25% (*P* < 0.001) and 50% (*P* = 0.004), but not at 75% (*P* = 0.385) and 100% (*P* = 0.257) of total exercise duration. For VL ΔRMS ⋅ M^-1^, significant differences between PLACEBO and NITRATE_tm_ were found at 25% (*P* = 0.032) and 50% (*P* = 0.013), but not at 75% (*P* = 0.170), and 100% (*P* = 0.169) of total exercise duration. Furthermore, a significant condition effect was shown for Q ΔRMS ⋅ M^-1^ (*F*_1,10_ = 6.59, *P* = 0.028, $\eta_{p}^{2}$ = 0.40). For Q ΔRMS ⋅ M^-1^, significant differences between PLACEBO and NITRATE_tm_ were found at 25% (*P* = 0.010) and 50% (*P* < 0.001), but not at 75% (*P* = 0.248) and 100% (*P* = 0.244) of total exercise duration (Figure 4). Absolute values of RMS ⋅ M^-1^ data across all muscles and time points are presented in Table 2.

**Table 2:** Electromyography and near-infrared spectroscopy recordings during time-matched conditions (*n* = 11).

			Time (% of total exercise duration)			
		Baseline	25	50	75	100
VM RMS ⋅ M^-1^						
	*PLACEBO*	0.038 ± 0.016	0.054 ± 0.023	0.062 ± 0.017	0.064 ± 0.017	0.061 ± 0.016
	*NITRATE_tm_*	0.035 ± 0.014	0.047 ± 0.020	0.050 ± 0.016	0.056 ± 0.015	0.055 ± 0.012
RF RMS ⋅ M^-1^						
	*PLACEBO*	0.065 ± 0.031	0.089 ± 0.045	0.096 ± 0.035	0.095 ± 0.038	0.094 ± 0.039
	*NITRATE_tm_*	0.069 ± 0.030	0.087 ± 0.043	0.088 ± 0.042	0.098 ± 0.047	0.096 ± 0.045
VL RMS ⋅ M^-1^						
	*PLACEBO*	0.036 ± 0.011	0.051 ± 0.018	0.054 ± 0.015	0.057 ± 0.015	0.055 ± 0.015
	*NITRATE_tm_*	0.036 ± 0.012	0.045 ± 0.016	0.047 ± 0.013	0.052 ± 0.012	0.050 ± 0.012
Q RMS ⋅ M^-1^						
	*PLACEBO*	0.044 ± 0.013	0.061 ± 0.023	0.070 ± 0.016	0.073 ± 0.018	0.073 ± 0.018
	*NITRATE_tm_*	0.045 ± 0.014	0.055 ± 0.022	0.060 ± 0.018	0.068 ± 0.019	0.069 ± 0.018
SmO_2_ (%)						
	*PLACEBO*	72.0 ± 7.3	33.4 ± 11.3	33.5 ± 14.0	32.8 ± 14.0	32.6 ± 12.3
	*NITRATE_tm_*	69.5 ± 9.8	36.9 ± 15.7	37.6 ± 19.2	35.6 ± 19.5	35.8 ± 21.2
THb (g ⋅ dL^-1^)						
	*PLACEBO*	12.7 ± 0.4	12.6 ± 0.5	12.6 ± 0.5	12.6 ± 0.5	12.6 ± 0.5
	*NITRATE_tm_*	12.6 ± 0.4	12.6 ± 0.4	12.6 ± 0.6	12.5 ± 0.6	12.6 ± 0.6

*Values are expressed as mean ± SD. RMS ⋅ M^-*1*^, root mean square of the electromyography signal normalized to the corresponding maximal M-wave; VM, vastus medialis; RF, rectus femoris; VL, vastus lateralis; Q RMS ⋅ M^-*1*^, RMS ⋅ M^-*1*^ was averaged across VM, RF, and VL to estimate the total activity of the quadriceps muscle; SmO_*2*_, muscle oxygen saturation; THb, total hemoglobin; NITRATE_*tm*_, time-matched nitrate condition*.

### Muscle Oxygenation During Exercise

There was no significant condition effect for ΔSmO_2_ (*F*_1,10_ = 5.09, *P* = 0.179, $\eta_{p}^{2}$ = 0.17) and ΔTHb (*F*_1,10_ = 0.14, *P* = 0.714, $\eta_{p}^{2}$ = 0.01).

Please note that a subsample analysis of participants who improved their exercise tolerance following NITRATE (*n* = 8) revealed that there was a significant condition effect for ΔSmO_2_ (*F*_1,7_ = 5.84, *P* = 0.046, $\eta_{p}^{2}$ = 0.46). Absolute values for SmO_2_ and THb during exercise are presented in Table 2.

### Perception of Effort and Leg Muscle Pain

Mean and end-exercise levels of perceived effort were significantly lower during NITRATE_tm_ compared to PLACEBO (*P* = 0.027, *d* = 0.47; *P* = 0.037, *d* = 0.36, respectively). Lower mean levels of leg muscle pain were documented during NITRATE_tm_ compared to PLACEBO (*P* = 0.031, *d* = 0.43). End-exercise levels of leg muscle pain were not significantly different between time-matched conditions (*P* = 0.066, *d* = 0.34). Absolute values of perceived effort and leg muscle pain are presented in Table 3.

**Table 3:** Perceptual responses during exercise (*n* = 11).

		Mean levels	End-exercise
Effort perception			
	*PLACEBO*	17 ± 2	19 ± 2
	*NITRATE_tm_*	16 ± 3^∗^	18 ± 3^∗^
Leg muscle pain			
	*PLACEBO*	5 ± 3	6 ± 3
	*NITRATE_tm_*	4 ± 2^∗^	5 ± 3

*Values are expressed as mean ± SD. The average of all ratings across the entire exercise duration is reported as mean levels of effort and leg muscle pain. End-exercise levels of effort and leg muscle pain refer to the last rating of effort and leg muscle pain before exercise termination. NITRATE_*tm*_, time-matched dietary nitrate condition. Significantly different between conditions: ^∗^P < 0.05*.

## Discussion

The present study was designed to provide further insight into the mechanistic basis for the improved exercise tolerance after dietary nitrate supplementation. We showed that dietary nitrate supplementation significantly improved exercise tolerance of the knee extensors during a high-intensity endurance task in two thirds of all subjects. Furthermore, by comparing time-matched exercise conditions with and without dietary nitrate supplementation, we found that dietary nitrate attenuated the development of muscle fatigue by reducing the exercise-induced impairments in contractile quadriceps function. Another important finding was that perception of effort and leg muscle pain was significantly lower following dietary nitrate supplementation.

### Exercise Tolerance and Muscle Fatigue

In the present study, we found that exercise tolerance during high-intensity OLDE was improved after a 5 days dietary nitrate supplementation as indicated by a significantly increased time-to-exhaustion. Improved tolerance to low and high-intensity whole-body endurance exercise was also found in healthy adults after acute and short-term (4–6 days) dietary nitrate supplementation (Bailey et al., 2010; Lansley et al., 2011; Thompson et al., 2014). In the present study, however, only 8 out of 12 participants improved their exercise tolerance following 5 days of dietary nitrate supplementation. Interindividual differences in the responsiveness to dietary nitrate supplementation were also found by others (e.g., Wilkerson et al., 2012; Coggan et al., 2018). It has been suggested that the participant’s training status (e.g., aerobic capacity), capillary density, endothelial NO synthase activity, and fiber type distribution can affect the impact of dietary nitrate supplementation on exercise performance (Jones, 2014b). Although we have not analyzed it, the interindividual variability in exercise performance could be further explained by differences in plasma [nitrate] and [nitrite] (Wilkerson et al., 2012; Coggan et al., 2018). As recently shown by Vanhatalo et al. (2018), this might be due to differences in the nitrate reducing capacity of oral microbiota. Those who have not improved their exercise tolerance following 5 days of supplementation with ∼6.5 mmol nitrate per day, might have benefited from a higher daily dosage or a longer supplementation period. Further research is warranted to elucidate if there are ‘non-responders’ to dietary nitrate supplementation or whether individualized dosing strategies are necessary to achieve an ergogenic effect.

By comparing time-matched conditions, we showed that dietary nitrate supplementation attenuated the development of quadriceps muscle fatigue as indicated by a significantly lower ΔMVT during NITRATE_tm_ (-42% ± 12%) compared to PLACEBO (-50% ± 11%). We found that ΔVA was not different between PLACEBO and NITRATE_tm_, suggesting that dietary nitrate does not significantly affect central factors of muscle fatigue during high-intensity OLDE. However, we have shown, for the first time, that dietary nitrate attenuates the development of quadriceps muscle fatigue mainly by reducing the impairments in contractile quadriceps function during high-intensity OLDE. This was indicated by a significantly lower ΔPS100 during NITRATE_tm_ (-36% ± 12%) compared to PLACEBO (-46% ± 8%). Increased dietary nitrate intake has been shown to improve vascular (Ferguson et al., 2013), metabolic (Bailey et al., 2010), and skeletal muscle function in response to exercise (Hernández et al., 2012; Haider and Folland, 2014). Any of the physiological alterations associated with dietary nitrate are capable of modulating skeletal muscle function during fatiguing exercise (Affourtit et al., 2015).

Bailey et al. (2010) have found that 4–6 days of dietary nitrate supplementation reduces the degradation of phosphocreatine (PCr) as well as the concomitant accumulation of adenosine diphosphate (ADP) and inorganic phosphate (P_i_). The latter is thought to be a main contributor to exercise-induced impairments in Ca^2+^ handling and myofibrillar Ca^2+^ sensitivity (Allen et al., 2008). Consequently, a lower intracellular [P_i_] associated with dietary nitrate supplementation seem to a plausible explanation for the reduced impairments in contractile function. On the one hand, data from Bailey et al. (2010) suggest that a reduction in the ATP cost of muscle force production might be responsible for the lower PCr degradation and accumulation of P_i_ following dietary nitrate supplementation. This assumption is supported by experiments in both humans (Haider and Folland, 2014; Whitfield et al., 2017) and mice (Hernández et al., 2012), showing that dietary nitrate enhances the contractile force production in response to low-frequency electrical stimulation. Hernández et al. (2012) have shown that the increased contractile force production results from an improved intracellular Ca^2+^ handling. On the other hand, there is data from experiments in rodents (Ferguson et al., 2013) and humans (Richards et al., 2018) suggesting that dietary nitrate increases muscle blood flow via local vasodilation, which in turn may improve oxygen delivery to the contracting muscles. It is generally well accepted that changes in O_2_ delivery to muscles alter intracellular metabolism, metabolite accumulation and ultimately contractile muscle function during exercise (Amann and Calbet, 2007). Since the rate of PCr hydrolysis and concomitant intracellular accumulation of P_i_ have been shown to be slower under conditions of increased O_2_ availability (Hogan et al., 1999), a higher O_2_ availability during exercise might have contributed to the reduced impairments in contractile quadriceps function following dietary nitrate supplementation. Improvements in NIRS-derived indices of muscle oxygenation following dietary nitrate supplementation were found in healthy adults (Bailey et al., 2009) and patients with peripheral arterial disease (Kenjale et al., 2011) during whole body endurance exercise. Bailey et al. (2009) have shown that deoxyhemoglobin peak amplitude, an estimate of muscle fractional O_2_ extraction, was significantly lower after beetroot juice consumption for 4–6 days when measured during moderate-intensity exercise. In the present study, however, there was no significant condition effect for ΔSmO_2_ of the VL during exercise. Although conclusions should be drawn with caution, a subsample analysis of those who have improved their time-to-exhaustion with dietary nitrate has revealed a significant condition effect. Consequently, it cannot be fully excluded that an improved SmO_2_ of the quadriceps muscle during exercise has contributed to the attenuated impairment in contractile function following dietary nitrate supplementation. Further studies are therefore needed to understand the exact causes for the reduced exercise-induced impairments in contractile function following dietary nitrate supplementation.

Furthermore, we found a significant condition effect for Q RMS ⋅ M^-1^ during exercise. Although only significant for the first half of the exercise protocol (see Figure 4), the exercise-induced increase in quadriceps muscle activity was lower during NITRATE_tm_ compared to PLACEBO. A rise in muscle activity in the course of a submaximal motor task at a constant power output is commonly interpreted as an increased recruitment of additional motor units to compensate for the progressive loss in contractile muscle function (Devries et al., 1982; Moritani et al., 1993). Since we found that the exercise-induced loss in contractile torque production is lower following dietary nitrate intake, less muscle activation might be required to ensure the same power output.

### Perceptual Responses During Exercise

In the present study, we found that NITRATE_tm_ resulted in lower mean and end-exercise levels of effort perception compared to PLACEBO, suggesting that dietary nitrate reduces the perception of effort during a high-intensity endurance task of the knee extensors. This finding is of particular importance since effort perception is thought to be a key-determinant of endurance performance (Marcora and Staiano, 2010). Based on the psychobiological model of exercise tolerance (Marcora, 2008), it has been stated that participants disengage from a task as a result of an effort-based decision. There is evidence suggesting that tolerance to high-intensity aerobic exercise in highly motivated athletes is predominantly limited by effort perception but not by the inability of muscles to generate the required power for the task (Marcora and Staiano, 2010). Our participants can be characterized as highly motivated since we (i) documented high levels of task motivation during each experimental condition, (ii) announced a monetary reward for the best three performers, and (iii) provided strong verbal encouragement during the task. Therefore, a reduced effort perception could be a significant contributor to the nitrate-induced improvements in exercise tolerance during high-intensity OLDE.

Although the exact physiological mechanisms underlying the perception of effort are still debated (Marcora, 2009), it is well accepted that neural processing of sensory signals in the brain is involved in effort perception (Noble and Robertson, 1996). Based on the corollary discharge model, it has been stated that effort perception reflects a centrally mediated feedforward mechanism in which an efference copy of the central motor command is sent from motor to sensory brain areas to enable a conscious awareness of processes associated with motor output (Poulet and Hedwig, 2007). During fatiguing contractions, the progressive rise in effort perception is thought to reflect the increase in central motor command which is necessary to compensate for the exercise-induced impairments in contractile muscle function in order to ensure adequate power output to maintain the task (de Morree et al., 2012). Consequently, the lower perception of effort following dietary nitrate supplementation could result from a reduced central motor command as a result of the preserved contractile function during exercise. By contrast, it has also been suggested that afferent feedback from working muscles contribute to the perception of effort (Amann et al., 2010). Although this assumption is highly debated (Marcora, 2009), it cannot be ruled out that an attenuated afferent feedback from the working muscles due to less metabolic disturbances in the periphery has contributed to the lower effort perception after dietary nitrate supplementation.

Although it is well accepted that perception of effort plays a crucial role in endurance performance (Marcora and Staiano, 2010), there is hardly any evidence for the impact of dietary nitrate on effort perception during endurance exercise. To the authors’ knowledge, there is only one study that recorded ratings of perceived exertion during work-matched submaximal exercise (Cermak et al., 2012). Cermak et al. (2012) have found that ratings of perceived exertion during submaximal constant-load cycling were not significantly affected by 6 days of dietary nitrate supplementation. However, since the authors have not reported the underlying definition of exertion, it is possible that participants’ rating included other exercise-related sensations than effort (e.g., discomfort and muscle pain) which are based on different neurophysiological mechanisms (Marcora, 2009). Therefore, it remains to be elucidated, if dietary nitrate supplementation also affects effort perception during submaximal, whole-body endurance tasks. Future studies investigating the mechanistic bases for the improved endurance performance following dietary nitrate supplementation should pay special attention on effort perception and its appropriate assessment [as recently proposed by Pageaux (2016)].

Furthermore, we found that 5 days of dietary nitrate supplementation resulted in lower mean levels of leg muscle pain during high-intensity OLDE, indicating that the participants had to tolerate less muscle pain in the course of exercise compared to PLACEBO. To our knowledge, there is no study to date assessing the impact of dietary nitrate on muscle pain during submaximal knee extension exercise. A previous study investigating patients with peripheral arterial disease has shown that dietary nitrate supplementation delays the onset of claudication pain during walking, which in turn resulted in an improved time-to-exhaustion (Kenjale et al., 2011). In healthy participants, exercise-induced muscle pain has also been proposed as an important factor in endurance performance (Mauger, 2013). Experimental evidence supporting this assumption is, however, scarce and often ambiguous (Stevens et al., 2018). Consequently, it cannot be ruled out that reductions in muscle pain perception contributed to the improved exercise tolerance.

While effort perception is likely centrally generated and seems to be independent of afferent feedback (Marcora, 2009), exercise-induced pain is thought to be related to feedback from nociceptive group III/IV muscle afferents about alterations associated with muscular contraction [e.g., increased intramuscular pressure, heat, high levels of metabolites, or deformation of tissue (Mense, 1993)]. As stated earlier, dietary nitrate supplementation has been shown to be associated with changes in local muscle perfusion, intramuscular metabolism, and reduced metabolite accumulation (Bailey et al., 2010). Given the fact that group III/IV afferents are sensitive to changes in metabolite concentration, it can be speculated that a reduced metabolite accumulation might attenuate the afferent feedback and thus lowers the perception of exercise-induced muscle pain. However, it should be noted that pain is not always directly related to the magnitude of the nociceptive signal, since pain is considered as subjective experience with a strong emotional component (Mauger, 2013). Further studies are necessary to better understand the impact of dietary nitrate on perception of exercise-induced muscle pain.

### Limitations

Although single-joint endurance exercise provides an appropriate model to investigate underlying determinants of endurance performance, whole-body endurance exercise (e.g., cycling, running, and walking) has the advantage to better mimic real world activities and sport events (Pageaux and Lepers, 2016). Given the fact that whole-body exercise requires a greater amount of muscle work with concomitant higher cardiorespiratory demands, there is a greater potential for systemic responses (e.g., hyperthermia, respiratory muscle fatigue, and arterial hypoxemia), which might affect the fatigability of the neuromuscular system (Sidhu et al., 2013). Therefore, the impact of dietary nitrate ingestion on muscle fatigue, perceptual responses, and its implications for exercise tolerance is currently limited to single-joint exercise.

In line with other studies using high-intensity OLDE (Pageaux et al., 2015, 2016), we found a large interindividual variability in exercise duration of the time-to-exhaustion test (Figure 2). Considering that both muscle fatigue and the effectiveness of dietary nitrate with regard to endurance performance are thought to be task-dependent (Enoka et al., 2011; Jones, 2014a), the observed variability in duration and intensity might have affected the impact of dietary nitrate on muscle fatigue development.

## Conclusion

The present findings extend the mechanistic basis for the improved exercise tolerance following dietary nitrate supplementation by investigating key-determinants of endurance performance. We showed, for the first time, that 5 days of dietary nitrate supplementation were associated with reduced levels of muscle fatigue compared to the time-matched placebo condition. Data indicate that the attenuated development of muscle fatigue following dietary nitrate ingestion was mainly due to lower exercise-induced impairments in contractile function (i.e., less peripheral fatigue). Therefore, dietary nitrate supplementation might be a promising approach to reduce muscle fatigue in situations (e.g., altitude) or populations (e.g., patients with peripheral arterial disease, type 2 diabetes, chronic heart failure) in which muscle fatigue is exacerbated and exercise tolerance is compromised. Another important finding of the present study was that dietary nitrate supplementation was accompanied by a lower effort perception during exercise. Based on the well-accepted corollary discharge model (Christensen et al., 2007; Poulet and Hedwig, 2007), we assume that the lower effort perception following dietary nitrate resulted from a lower rise in central motor command as an adjustment to the preserved contractile function compared to PLACEBO. We conclude that an attenuated development of muscle fatigue as well as lower levels of perceived effort and muscle pain have contributed to the improved exercise tolerance following dietary nitrate supplementation.

## Ethics Statement

The study was approved by the university ethics committee and was conducted according to the Declaration of Helsinki. All subjects were informed about possible risks and discomfort associated with the investigations prior to giving their written consent to participate.

## Author Contributions

FH and MB designed the study and collected, analyzed, and interpreted the data. FH wrote the manuscript. MB, SB, TM, and VZ contributed to writing, reviewing, and editing of the manuscript. All authors approved the final version of the manuscript.

## Conflict of Interest Statement

The authors declare that the research was conducted in the absence of any commercial or financial relationships that could be construed as a potential conflict of interest.
